# The first complete mitogenome of red-bellied parrot (*Poicephalus rufiventris*) resolves phylogenetic status within Psittacidae

**DOI:** 10.1080/23802359.2018.1437818

**Published:** 2018-02-10

**Authors:** Subir Sarker, Shubhagata Das, Seyed A. Ghorashi, Jade K. Forwood, Karla Helbig, Shane R. Raidal

**Affiliations:** a Department of Physiology, Anatomy and Microbiology, School of Life Sciences, La Trobe University, Melbourne, Australia;; b School of Animal and Veterinary Sciences, Faculty of Science, Charles Sturt University, Albury, Australia;; c School of Biomedical Sciences, Charles Sturt University, Albury, Australia

**Keywords:** Avian mtDNA, mitogenome phylogeny, family Psittacidae, *Poicephalus rufiventris*

## Abstract

This paper describes the genomic architecture of a complete mitogenome from a red-bellied parrot (*Poicephalus rufiventris*). The mitogenome sequence was circular and 15,524 bp in length. Compared to other Psittacidae species, the genome encoded a conserved structure consisting of 13 protein-coding genes (PCGs), two rRNA genes, 21 tRNA genes, and two control regions, however, the mitogenome was missing a part of control region and a tRNA-Leu. The lengths of 12S and 16S ribosomal RNA were 975 bp and 1569 bp, respectively. The overall base composition of the mitogenome of *P. rufiventris* was dominated by higher AT (51.6%) than GC (48.4%) content. The complete mitogenome sequence determined in this study would be useful to track the deeper evolutionary history and the conservation of *P. rufiventris.*

The red-bellied parrot (*Poicephalus rufiventris*) has an extremely large range, and hence the population trend appears to be stable (BirdLife International 2016). The subfamily Psittacinae comprise a genus *Poicephalus*, which in contrast to monotypic Psittacus, is the most species-rich and widely distributed in Africa (Urantówka et al. 2017). *Poicephalus* is morphologically diverse (Urantówka et al. 2017), and several of its species (*fuscicollis*, *gulielmi*, *senegalus*, *flavifrons*, and *meyeri*) are further divided into subspecies (Gill and Donsker 2017). Therefore, this parrot offers an interesting possibility to study mechanisms of speciation and emergence of new lineages, whilst there are still major uncertainties in the position of many avian species including *P. rufiventris* due to a lack of abundant mitochondrial (mt) datasets. Moreover, the complete mitogenomes could play a major role to understand the origin, evolution, and divergence time of speciation, as well as influencing conservation and management decisions of species (Eo et al. 2010). This paper describes the genomic architecture of a complete mitogenome of *P. rufiventris,* which will further strengthen our understanding of the species diversity, host phylogeny, and ecological diversity of the species.

The liver tissue used in this study was sourced from a male red-bellied parrot (*Poicephalus rufiventris*) in a captive aviary flock (year of sampling: 2014), which was stored in appropriate conditions by the Veterinary Diagnostic Laboratory (VDL), Charles Sturt University under the accession number CS14-3247. Animal sampling was obtained in accordance with approved guidelines set by the Australian Code of Practice for the Care and Use of Animals for Scientific Purposes (1997) and approved by the Charles Sturt University Animal Ethics Committee (Research Authority permit 09/046), and the total genomic DNA was extracted using an established protocol (Sarker et al. 2015, 2016; Das et al. 2017). The genomic library preparation and sequencing was performed according to the published protocol (Das et al. 2017; Sarker, et al. 2017). Briefly, the paired-end library was prepared with an insert size of 150 bp using the Illumina paired-end sample preparation kit (Illumina, San Diego, CA, USA) according to the manufacturer's instructions. A HiSeq4000 sequencing platform (Novogene, Hong Kong, China) was used, which was generated approximately 10.52 million sequence reads from the genomic DNA of red-bellied parrot. The raw datasets were trimmed to pass the quality control based on PHRED score or per base sequence quality score, and the assembly of the mitochondrial genome was conducted according to the established pipeline in CLC Genomics workbench 9.5.4 under La Trobe University Genomics Platform (Sarker et al. 2017; Sarker, et al. 2017). Annotation was performed with MITOS (Bernt et al. 2013), and protein coding ORFs were further assessed using the CLC Genomics Workbench (version 9.5.4).

The complete mitogenome sequence of *P. rufiventris* had a circular genome of 15,524 bp, containing 13 protein-coding genes (PCGs), two rRNA genes, 21 tRNA genes and two control regions, however, the mitogenome is missing a part of control region and a tRNA-Leu. The contents of A, T, C and G were 29.7, 21.9, 33.8, and 14.6%, respectively. AT and GC contents of this complete mitogenome was 51.6 and 48.4%, respectively. The proportion of coding sequences with a total length of 11,085 bp (71.37%), which encodes 3695 amino acids, and all PCGs started with Met. The lengths of 12S and 16S ribosomal RNA were 975 bp and 1569 bp, respectively. The gene arrangement was similar to the complete mitochondrial genome of other Psittacidae species.

Phylogenetic analysis was performed using complete mitogenome sequence of a *P. rufiventris* determined in this study with the other mitogenome sequences obtained from species belonging to the family Psittacidae available in GenBank. The sequences were aligned using the MAFFT L-INS-i algorithm (Katoh et al. 2002), and the maximum likelihood (ML) tree with 1,000 non-parametric bootstrap resamplings were generated using CLC Genomics workbench 9.5.4. As highlighted in Figure 1, the mitogenome sequence of *P. rufiventris* generated a monophyletic clade with a grey parrot (*Psittacus erithacus*; GenBank accession no. KM611474*)* also native to Africa (Eberhard and Wright 2016), and demonstrated a >82% pairwise nucleotide identity between them. We concluded that the complete mitogenome of *P. rufiventris* will be a useful database among the genus *Poicephalus* to study further host-phylogenetic relationship of Psittacidae species, and suggest this may be an implication for the conservation of the species.

**Figure 1.**
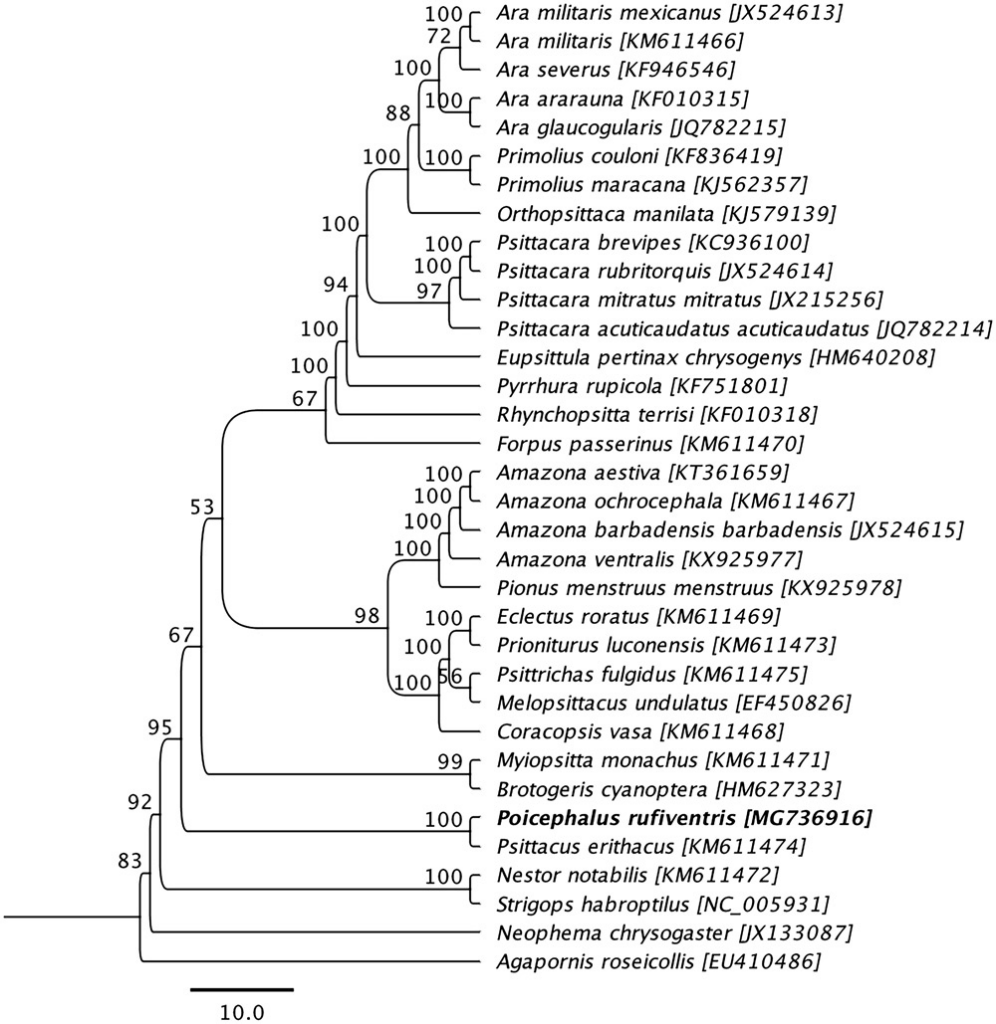
Maximum likelihood phylogenetic tree to infer host-phylogeny relationship among Psittacidae family. ML-tree was constructed using complete mitogenome sequences of the species belonging to the Psittacidae family. The new complete mitogenome of *P. rufiventris* is highlighted by bold font.
